# Exposure to Synthetic Endocrine-Disrupting Chemicals in Relation to Maternal and Fetal Sex Steroid Hormones: A Scoping Review

**DOI:** 10.1007/s40572-024-00455-6

**Published:** 2024-07-22

**Authors:** Megan C. Hansel, Abigail M. Rosenberg, Carolyn W. Kinkade, Camila Capurro, Zorimar Rivera-Núñez, Emily S. Barrett

**Affiliations:** 1 Department of Biostatistics and Epidemiology, Rutgers School of Public Health, Piscataway, NJ USA; 2 Department of Obstetrics and Gynecology, University of Rochester Medical Center, 601 Elmwood Ave., Rochester, NY 14642 USA; 3 Environmental and Occupational Health Sciences Institute, Rutgers University, 170 Frelinghuysen Rd, Piscataway, NJ 08854 USA

**Keywords:** Synthetic chemicals, Endocrine-disrupting chemicals, Sex steroid hormones, Estrogens, Androgens, Pregnancy

## Abstract

**Purpose of Review:**

Many synthetic endocrine-disrupting chemicals (EDCs) are ubiquitous in the environment and highly detected among pregnant people. These chemicals may disrupt maternal and/or fetal sex steroid hormones, which are critical to pregnancy maintenance and fetal development. Here, we review the epidemiological literature examining prenatal exposure to common synthetic EDCs in relation to maternal and fetal sex steroid hormones.

**Recent Findings:**

We performed a literature search using PubMed, SCOPUS, and Embase, ultimately identifying 29 articles for full review. Phenols, parabens, and persistent organic pollutants generally showed inverse associations with androgens, estrogens, and progesterone. Phthalates and per-and polyfluoroalkyl substances tended to be inversely associated with progesterone, while evidence regarding androgens and estrogens was mixed. Inconsistent, but noteworthy, differences by fetal sex and timing of exposure/outcome were observed.

**Summary:**

Overall, the literature suggests EDCs may disrupt maternal and fetal sex steroid activity, though findings are mixed. Given the pervasive, high-volume production of these synthetic chemicals and the critical functions sex steroid hormones play during gestation, additional research is warranted.

**Supplementary Information:**

The online version contains supplementary material available at 10.1007/s40572-024-00455-6.

## Introduction

During pregnancy, the endocrine system supports pregnancy maintenance and fetal development. Sex steroid hormones, including estrogens, progesterone, and androgens, are important to the development of essential systems and functions (e.g., growth, reproduction), but may also be vulnerable to disruption by exogenous exposures [1]. During pregnancy, estrogens serve key functions such as increasing blood flow to the uterus, stimulating tissue and uterine growth, and promoting the development of fetal organs [2–4]. In early pregnancy, most estrogen is produced by maternal organs (e.g., ovaries, adipose tissue), however as pregnancy progresses, the placenta takes over as the primary producer [4]. Similar to estrogens, progesterone is predominately produced by the placenta during pregnancy [4]. Progesterone prevents premature uterine contractions and modulates the mother’s immune response [4, 5]. In comparison to estrogens and progesterone, androgen activity in pregnancy has been less extensively studied. Key androgens include total testosterone (TT) and free testosterone (fT), which may derive from the ovaries and adrenal gland [6]. Additionally, testosterone is aromatized into estrogen by aromatase, which is highly expressed in the placenta [6]. Androgens may play a role in sexual differentiation [7]. Several epidemiological studies have linked altered sex steroid concentrations in maternal circulation or cord blood to pregnancy complications, as well as child outcomes including growth and neurodevelopment [8–10]. These epidemiological studies are supported by toxicological studies indicating adverse outcomes associated with sex steroid hormone manipulation [11–13]. Ultimately, the disruption of sex steroid hormone pathways during pregnancy may have important implications for maternal and child health and has been the subject of a growing scientific literature [14].

In the modern world, a major source of endocrine disruption is through exposure to synthetic endocrine disrupting chemicals (EDCs)- compounds that interfere with the body’s hormone activity [15]. Over 1,000 chemicals have proven or suspected endocrine disrupting properties, many of which are widely found in pharmaceuticals, agricultural products, food, drinking water, and consumer goods [16]. As a result, many EDCs are widely found in the environment and have high levels of detection in humans, including pregnant people [17–19]. As the production of chemicals increases, it is vital to understand their impact on health, particularly during pregnancy. Recognizing the central role of hormone activity in maternal-fetal health, the objective of this review was to summarize the current epidemiologic literature on prenatal exposure to synthetic chemicals in relation to maternal and fetal sex steroid hormones. The classes of synthetic chemicals included in this review were chosen based on their designation as common EDCs by the Endocrine Society and included phenols, parabens, phthalates, poly- and perfluoroalkyl substances (PFAS), polychlorinated biphenyls (PCBs), flame retardants, pesticides, and dioxins [15].

## Methods

### Search Strategy

We performed a scoping review to evaluate the current epidemiological literature examining prenatal synthetic EDCs in relation to sex steroid hormones in pregnant people and their fetuses. This review was conducted according to the PRISMA Extension for Scoping Reviews. The PRISMAScR checklist is provided in Supplemental Table 1. A protocol for this review was registered in PROPSPERO (ID CRD42023440353). Studies were identified through searches in PubMed, SCOPUS, and Embase. Major concepts for searching were synthetic chemicals and maternal and fetal sex steroid hormones. A medical librarian consulted on the initial search strategy. Search terms and strategies are shown in Supplemental Table 2.

### Study Screening and Selection

We reviewed original epidemiologic studies in pregnant people relating maternal exposure to one or more EDCs of interest (i.e., phenols, parabens, phthalates, PFAS, PCBs, flame retardants, pesticides, and dioxins) to any of the outcomes (maternal or fetal sex steroid hormones: testosterone, free testosterone, estrone, estriol, estradiol, and progesterone). Following our search, articles were exported and duplicates were removed in Rayyan. Next, we screened the article titles and abstracts for relevance in Rayyan, which enabled the screenings to be independent and blinded. Each title and abstract were reviewed independently by two reviewers to determine whether the full text should be reviewed. Both reviewers examined the full text and made an independent determination about whether it should be included. Any discrepancies were resolved through discussion, including with additional co-authors as needed. We retrieved the full-text version of the remaining articles and assessed eligibility. Study inclusion criteria were based on our PECO statement (Table 1) and were as follows: [1] published in English; [2] human participants; [3] publication dates from January 1st, 2000 to July 5th, 2023 (the date when the initial search was performed); [4] any race, ethnicity, socioeconomic status, or geographical location; [5] primary research studies; [6] examined maternal exposure to a synthetic chemical (phenols, parabens, phthalates, PFAS, PCBs, flame retardants, pesticides, or dioxins) in relation to maternal or fetal sex steroid hormones (testosterone, free testosterone, estrone, estriol, estradiol, or progesterone). Searches were limited to after the year 2000 in an effort to highlight the most current literature. Studies were excluded if they did not meet these criteria, if the synthetic chemical exposure was examined in a fetal (rather than maternal) biomarker (e.g., cord blood and amniotic fluid), or if the exposure or outcome was measured in a non-preferred matrix. Preferred maternal chemical matrices are as follows: phenols- urine; parabens- urine; phthalates- urine; PFAS- blood; PCBS- blood; flame retardants (polybrominated diphenyl ethers)- blood; organophosphate pesticides- urine; organochlorine pesticides- blood; dioxins- blood. Acceptable hormone matrices included maternal serum, maternal plasma, maternal urine, cord plasma, cord serum, or cord blood. Primary searchers were run in PubMed, SCOPUS, and Embase on July 5, 2023. The search was rerun in PubMed on January 15, 2024 to capture additional papers that had been published since the original search was completed. Following screening, data charting was done independently by one reviewer. For quality assurance and consistency, each table was reviewed by a second author, after which all tables were edited for consistency by the first author as needed. For each study, we extracted the study sample and location, study design, exposure measures and assay, exposure source and timing, outcome measures and assay, outcome source and timing, and main findings. This information is presented in Tables 2 and 3, which examine non-persistent and persistent chemical exposures, respectively. Compounds that were the focus of five or more papers (e.g., phthalates, PFAS), were discussed in a discrete sub-section. Parabens and phenols were discussed together, as parabens are considered phenolic compounds [20]. The remaining chemical classes with limited literature (< 5 studies), were synthesized within chemical class for discussion. These included PCBs, flame retardants, pesticides, and dioxins. A critical appraisal of the evidence including sources of bias was not performed as part of this Scoping review.

**Table 1:** PECO statement

Population	Exposure	Comparators	Outcomes
Pregnant people and their fetuses	Maternal exposure to synthetic chemicals including phenols, parabens, phthalates, PFAS, PCBs, flame retardants, pesticides or dioxins measured in a preferred matrix (Phenols- urine; Parabens- urine; Phthalates- urine; PFAS- blood; PCBs- blood; Polybrominated Diphenyl Ethers- blood; Organophosphate Pesticides- urine; Organochlorine Pesticides- blood; Dioxins- blood)	Pregnant people with lower or no exposure	Maternal and/or fetal sex steroid hormones including testosterone, free testosterone, estrone, estriol, estradiol, or progesterone measured in a preferred matrix (maternal serum, maternal plasma, maternal urine, cord plasma, cord serum, cord blood)

**Table 2:** Summary of epidemiological studies examining the relationship of maternal non-persistent chemical exposures with maternal and/or fetal sex steroid hormones

First author (year)	Study sample and location	Study design	Exposure measures and assay	Exposure source and timing	Outcome measures and assay	Outcome source and timing	Main findings
*Phenols*							
Aker et al. [33]	106 pregnant people from the PROTECT cohort in Puerto Rico, U.S.	Cohort*	BPA, TCS, BP-3, 2,4-DCP, and 2,5-DCP measured using HPLC-MS/MS	Mid-pregnancy maternal urine (Visit 1: 16–20; Visit 3: 24–28 gestation weeks)	P and E2 measured using a chemiluminescence immunoassay	Mid-pregnancy maternal serum (Visit 1: 16–20; Visit 3: 24–28 gestation weeks)	-No significant associations between phenols and E2 -All phenols non-significantly, inversely associated with P -At visit 1, non-significant inverse associations between all phenols and E2 and P -At visit 3, mixed direction of association, all non-significant
Aker et al. [34]	602 Pregnant people from the PROTECT cohort in Puerto Rico, U.S.	Cohort*	2,4-DCP, 2,5-DCP, BPA, BPS, BPF, TCS, and TCC measured using HPLC-MS/MS	Mid-pregnancy maternal urine (Visit 1: 16–20; Visit 3: 24–28 gestation weeks)	P and T measured using chemiluminescence immunoassay; E3 measured using ELISA	Mid-pregnancy maternal serum (Visit 1: 16–20; Visit 3: 24–28 gestation weeks)	-No statistically significant results in mixed models -At 24–28 weeks, BPA associated with 17% lower T (%Δ -17.37; 95% CI: -26.7, -6.87) -At 24–28 weeks, TCS associated with non-significantly higher T, P, and E3
Banker et al. [35]	56 mother-child pairs from the MMIP cohort in Michigan, U.S.	Cohort*	BPA, BPF, BPS, 2,4-DCP, 2,5-DCP, BP-3, TCC, and TCS measured using ID-LC-MS/MS	First trimester maternal urine	E1, E2, E3, T, and P measured using LC-MS	Maternal first trimester and term plasma; Cord plasma collected following delivery	-Negative association between BP-3 and first trimester E2 (*P* < 0.05) -Positive association between BPA and E3 in mothers carrying male fetuses (*P* < 0.05)
Jacobson et al. [36]	139 pregnant people from the CHES study in New York, USA	Cohort*	BPA; BPAF, BPAP, BPB, BPF, BPP, BPS, and BPZ measured using HPLC-MS/MS	Early (≥ 5-<18 weeks) and mid-pregnancy (≥ 18-<25 weeks) maternal urine	P measured using GC-MS/MS	Mid-pregnancy (≥ 18-<25 weeks) maternal serum	ΣBisphenols associated with non-significantly lower P (%Δ:-4.8; 95%CI: -10.4, 1.1)
Liu et al. [37]	100 mother-male infant pairs from two towns in China (one polluted, one cleaner)	Cross-sectional	BPA, 4-NP, and 4-tOP measured using GC-MS/MS	Late third trimester maternal urine	T and E2 Measured using RIA	Cord serum at delivery	-BPA inversely associated with T (β:-31.1ng/dl, 95%CI: -53.07, -9.11) -Compared to Q1 BPA, those in Q4 BPA had significantly lower T (β:-180ng/dl, 95% CI:-333.5, -26.2) -No significant relationship between BPA and E2
Wang et al. [38]	537 pregnant people from the LWBC cohort in Shandong Province, China	Cohort*	TCS measured using UPLC-MS/MS	Maternal urine at delivery	T and E2 measured using RIA	Cord serum at delivery	-Infants in the > 0.6 µg/L TCS group had higher T (β:0.09ng/mL, 95%CI:0.02, 0.16) compared to those < LOD; similar association with continuous TCS (β:0.04, 95%CI:0.00, 0.07) -Significant association between T and TCS in mothers carrying male but not female infants (male: β:0.06ng/mL, 95%CI:0.01, 0.12) -Mothers of males in the 0.1–0.6 µg/L TCS group had lower E2 (β:-0.09pg/mL, 95%CI:-0.15, -0.03) compared to those < LOD, mothers of females in the 0.1–0.6 µg/L group had higher E2 (β:0.06 pg/mL, 95%CI:-0.00, 0.13) compared to those < LOD
Guo et al. [39]	499 mother-child pairs Sheyang County, Jiangsu Province, China	Cohort*	BPA, TCS, BP-3, 4-tOP, 2,4-DCP, and 2,5-DCP measured using GC-MS/MS	Maternal urine at delivery	T and E2 Measured using automatic chemiluminescence immunoassay system	Cord serum at delivery	-In generalized linear models, among participants carrying males, a one-unit increase in BPA associated with lower E2 (β:-0.05; 95%CI:-0.11, -0.001); among participants carrying females TCS associated with lower T (β:-0.08; 95%CI:-0.14, -0.01) and E2 (β:-0.07, 95%CI:-0.14, 0.002) -In elastic net regression models, in women carrying female fetuses, TCS associated with lower T (β:-0.08; 95%CI:-0.14, -0.01) -In BKMR models, no significant associations between phenol mixtures and cord hormones
Li et al. [40]	851 pregnant people from Wuhan, China	Cohort*	BPA measured using UHPLC coupled with TQMS	Maternal urine in trimesters 1, 2, and 3	E1, E2, E3 measured using UHPLC coupled with TQMS	Maternal urine in trimesters 1, 2, and 3	-In each trimester, compared to Q1 BPA, those in the upper quartiles (Q2, Q3, and Q4) of BPA exposure had lower E1, E2, and E3, except for positive associations between Q4 BPA and E3 in each trimester -Continuous BPA had significant nonlinear associations with first trimester E2 and E3, second trimester, E1, E2, and E3, and third trimester E1 and E3 (< 0.05); overall associations were significant for E1, E2, and E3 in the first and second trimester -Associations between quartile BPA exposure and estrogens were generally inverse for both mothers carrying male and female fetuses, but significant associations were mostly observed among those carrying males
Zhang et al. [41]	726 mother-child pairs from the SMBCS cohort in Sheyang County, China	Cohort*	TCS measured using GC-MS/MS	Maternal urine at delivery	E2 and T measured using electrochemiluminescence assays	Cord serum at delivery	-In mediation models with TCS as the mediator and ponderal index as the outcome, TCS was associated with lower E2 (β:-6.78nmol/L, 95%Credible Interval:-6.29, -7.27) and T (β:-3.48pg/mL, 95%Credible Interval:-3.04, -3.91) -TCS as the mediator and head circumference was the outcome, TCS was associated with lower E2 (β:-5.29nmol/L, 95%Credible Interval:-4.79, -5.79) and T (β:-9.57pg/mL, 95%Credible Interval:-9.02, -10.12)
*Parabens*							
Aker et al. [33]	106 pregnant people from the PROTECT cohort in Puerto Rico, U.S.	Cohort*	BuPB, MBP and PPB measured using HPLC-MS/MS	Mid-pregnancy maternal urine (Visit 1: 16–20; Visit 3: 24–28 weeks gestation)	E2 and P measured using chemiluminescence immunoassay	Mid-pregnancy maternal serum (Visit 1: 16–20; Visit 3: 24–28 weeks gestation)	-In mixed effects models, IQR increase in BuPB was associated with 8.46% lower E2 (95%CI:-16.92, 0.00) -All metabolites except BuPB associated with non-significantly lower P across pregnancy -The associations between MPB and PPB with E2 were inverse at weeks 16–20, but positive at weeks 24–28
Aker et al. [34]	602 pregnant people from the PROTECT cohort in Puerto Rico	Cohort*	EPB, MPB, BuPB, and PPB measured using HPLC-MS/MS	Mid-pregnancy maternal urine (Visit 1: 16–20; Visit 3: 24–28 weeks gestation)	P and T measured using a chemiluminescence immunoassay; E3 measured using ELISA	Mid-pregnancy maternal serum (Visit 1: 16–20; Visit 3: 24–28 weeks gestation)	-BuPB, MPB, and PPB associated with lower T across pregnancy -All parabens associated with non-significantly lower E2
Banker et al. [35]	56 mother-child pairs from the MMIP cohort in Michigan, U.S.	Cohort*	BuPB, EPB, MPB, and PPB measured using ID-LC-MS/MS	First trimester maternal urine	E1, E2, E3, T, and P measured using LC-MS	Maternal first trimester and term plasma; Cord plasma collected following delivery	-Positive, non-significant association between EPB and maternal term T among mothers of males (β:0.9nmol/L, BH: 0.69), but negative, inverse association among mothers of females (β:-0.25nmol/L, BH:0.02) -Positive association between BuPB and cord P (β:0.35nmol/L, BH:0.10)
Guo et al. [39]	499 mother-child pairs Sheyang County, Jiangsu Province, China	Cohort*	MEP, EPB, PPB, BuPB, and BEP measured using GC-MS/MS	Maternal urine at delivery	T and E2 measured using automatic chemiluminescence immunoassay system	Cord serum at delivery	No significant associations between parabens and T or E2
*Phthalates*							
Johns et al. [42]	106 pregnant people from the PROTECT cohort in Puerto Rico	Cohort*	MEHP, MBP, MEHHP, MEOHP, MECPP, MCPP, MCOP, MCNP, MBzP, MiBP, and MEP measured by HPLC–IDMS	Mid-pregnancy maternal urine (Visit 1: 16–20; Visit 3: 24–28 gestation weeks	E2 and P measured with chemiluminescence	Mid-pregnancy maternal serum (Visit 1: 16–20; Visit 3: 24–28 gestation weeks	-In LMMs, an IQR increase in MEP was significantly associated with a 10.6% lower P (95%CI:-17.6, -2.84) -In LMMs, no significant associations with E2
Cathey et al. [18]	677 pregnant people from the PROTECT cohort in Puerto Rico	Cohort	MEHP, MEHHP, MEOHP, MECPP, MEP, MBP, MBzP, MIBP, MHiBP, MCPP, MCNP, MCOP, MHBP, MINP, MONP, MHiNCH, MCOCH, MECPTP, and MEHHTP measured with HPLC–IDMS	Mid-pregnancy maternal urine (Visit 1: 16–20; Visit 3: 24–28 weeks gestation)	P and T measured using a chemiluminescence immunoassayy; Estriol measured using ELISA	Mid-pregnancy maternal serum (Visit 1: 16–20; Visit 3: 24–28 weeks gestation)	-No significant associations between phthalates and E3 -In LMMs, an IQR increase in MCOP was associated with 9.85% lower P (95%CI:-17.0, -2.03); MEP was associated with lower T (∆%:-14.5, 95%CI:-24.3, -3.42); MHBP was associated with higher T (∆%:4.71, 95%CI:0.27, 9.35) -At visit 1, MCOP was associated with higher T (∆%:16.5, 95%CI:3.83, 30.7) and lower P (∆%:-10.8, 95%CI:-19.4, -1.19); MCNP was associated with a lower P/E3 ratio (∆%:-4.37, 95%CI:-8.09, -0.49) -At visit 3, MEP was associated with lower T (∆%:-18, 95%CI: -30.3, -3.57); MEHHTP with lower P (∆%:-13.1, 95%CI:-22.3, -2.75); MCOP and with lower P/E3 ratio (∆%:-14.0, 95%CI:-25.3, -1.02)
Jacobson et al. [36]	139 pregnant people from the CHES study in New York City, USA	Cohort	MMP, MEP, MBP, MIBP, MIPRP, MPEP, MCHP, MECPTP, MEOHP, MEHHP, MCMHP, MnOP, MCPP, MCHPP, MCOP, MiNP, MCNIP, MHpP, MBzP, MHXP, and MEHP measured using HPLC-MS/MS	Maternal urine in early (< 18 weeks) and midpregnancy (≥ 18-<25 weeks	P measured with HPLC GC-MS	Mid-pregnancy maternal serum (≥ 18-<25 weeks)	-∑DnOP (∆%:-8.1 95%CI:-15.2, -0.4). and ∑DiNP (∆%:-7.7 95%CI: -13.3, -1.7) associated with lower P -∑DEHP was non-significantly, inversely associated with P
Banker et al. [35]	56 pregnant people from the MMIP cohort in Michigan, USA	Cohort*	MECPP, MEHHP, MEHP, MEOHP, MIBP, MBP, MBzP, MCNP, MCPP, MCOMHP, MEP, and MINP measured with ID-LC/MS/MS	First trimester maternal urine	E1, E2, E3, T, and P measured with LC-MS/MS	Maternal first trimester and term plasma; Cord plasma collected following delivery	-MBP inversely correlated with maternal term and cord plasma E1 levels. No other significant correlations -The association between E3 and MEHP was more negative in mothers with a high BMI carrying female (BH:0.05)
Sathyanarayana et al. [45]	591 pregnant people from the TIDES cohort in California, Minnesota, New York, and Washington, USA	Cohort*	MEHP, MEHHP, MEOHP, MECPP, MEP, MIBP, MBzP, MBP, MCNP, and MCOP measured with reversed HPLC-IDMS	Early pregnancy maternal urine	TT, E1 and E2 measured with LC-MS/MS and fT measured with equilibrium dialysis	Early pregnancy maternal serum	-Generally, phthalate metabolites positively associated with estrogens -Positive associations between E1 and MiBP (∆%:30.38, 95%CI:9.02, 55.96), MEHP (∆%:28.23, 95%CI:9.85, 49.69), and MEOHP (∆%:28.23, 95%CI:9.85, 49.69) -Positive associations between E2 and MiBP (∆%:29.99, 95%CI:12.20, 50.59), MEHP (∆%:24.97, 95%CI:10.00, 41.97), MEOHP (∆%:24.97, 95%CI:10.00, 41.97), and MBzP (∆%:14.66, 95%CI:1.30, 29.75) -Significantly lower fT associated with MCNP (∆%:-12.40, 95%CI:-22.48, -1.03) and MECPP (∆%:-12.30, 95%CI:-21.17, -2.43) -Weak inverse trends between fT and TT and DEHP metabolites -Models assessing interaction with fetal sex were insignificant
Pacyga et al. [44]	439 pregnant people from the I-KIDS study in Illinois, USA	Cohort*	MEHP, MEHHP, MEOHP, MECPP, MiNP, MCOP, MCNP, MCPP, MBzP, MEP, MBP, MHBP, MIBP, MHiBP, MHiNCH, MCOCH, MONP, MEHHTP, and MECPTP measured with ID-LC/MS/MS	Maternal urine collected at 8–15, 14–22, 19–28, 25–33, and 32–40 weeks gestation. Samples pooled for analysis	∑estrogens (E1, E2, E3, 16α-hydroxyestrone, 2-hydroxyestrone, 2-methoxyestrone, 4-hydroxyestrone, and 4-methoxyestrone) and ∑Testosterone (T and 5α-dihydrotestosterone) measured with HPLC-MS/MS	∑ of three maternal first-morning urine samples collected at 8–15, 25–33, and 32–40 weeks gestation	-Two-fold increases in ∑DEHP (∆%:7.2, 95%CI:3.9, 10.6), MCNP (∆%:3.2, 95%CI:0.4, 6.1), MCPP (∆%:4.4, 95%CI:1.7, 7.1), ∑Plastics (∆%:5.4, 95%CI:2.3, 8.7), ∑DiNCH (∆%:3.0, 95%CI:1.0, 5.1), ∑DEHTP (∆%:3.7, 95%CI:1.2, 6.2), and ∑DBP (∆%:4.6 95%CI:1.3, 8.1) positively associated with ∑Estrogens -∑Testosterone significantly higher in women at the upper quartiles of ∑DEHP (Q3), MCPP (Q3), ∑Plastics (Q3), MEP (Q3 and Q4), ∑DiBP (Q2), and ∑PCP (Q2) compared to those in Q1; a two-fold increase in MEP was associated with 9.3% higher T (95%CI:1.8, 17.5) -Estrogen/Androgen ratio significantly lower in women at the upper quartiles of ∑DEHP, MCPP, MEP, and ∑PCP compared to those in Q1 - In linear models, a two-fold increase in MEP (∆%:-10.0, 95%CI:-16.4, -3.0) and ∑PCP (∆%:-10.1, 95%CI:-18.8, -0.6) associated with a lower estrogen/androgen ratio
Sathyanarayana et al. [45]	180 pregnant people from the SFFI cohort study in California, Minnesota, and Missouri, USA	Cohort*	MEP, MBP, MMP, MBzP, MIBP, MEHP, MEHHP, and MEOHP measured by HPLC-IDMS	Maternal second or third trimester urine	T and E2 measured with LC-MS/MS and fT measured with equilibrium dialysis	Maternal second or third trimester serum	-Among women carrying male fetuses, MBP associated with lower TT (β:-0.20, 95%CI:-0.39, -0.01), fT (β:-0.21, 95%CI:-0.42, 0.004), and E2 (β:-0.002, 95%CI:-0.18, 0.17); ∑DEHP associated with lower TT, (β:-0.15, 95%CI:-0.26, -0.04) and fT (β: -0.15, 95%CI:-0.27, -0.03) -Among women carrying female fetuses, MEP associated with increased TT (β:0.09, 95%CI:0.003, 0.17) and fT (β:0.10, 95%CI: -0.01, 0.19), but lower E2 (β:-0.03, 95%CI: -0.10, -0.04) -Did not examine associations in combined cohort
Lin et al. [46]	155 pregnant people from Taiwan	Cohort	MMP, MEP, MBP, MBzP, MEHP, MEOHP, MEHHP and ∑DEH measured with LC-ESI-MS/MS	Third trimester maternal urine	fT and E2 measured with RIA	Cord serum at birth	-In mothers carrying female fetuses, ΣDEHP significantly associated with lower fT (β:-0.23 ng/dL^− 1^, *p* < 0.05) and FT/E2 (β:-0.22, *p* < 0.05) -No significant results among mothers carrying male fetuses
Hlisnikova et al. [48]	90 pregnant people from the PRENATAL cohort in Slovakia	Cohort*	MMP, MEP, MiBP, MBP, MHiBP, OH-MnBP, MnPeP, MCHP, MBzP, MEHPP, MEOHP, MECPP, MiNP, OH-MiNP, cx-MiNP, oxo-MiNP, MnOP, ∑DiBP, ∑DnBP, ∑DEHP, ∑DiNP, ∑LMWP, and ∑HMWP measured using HPLC-MS/MS	Maternal urine from early pregnancy	E2 and T measured with electrochemiluminescence	Maternal serum in early pregnancy	-Significant positive associations between OH-MiNP and E2 (β:0.24pg/mL; 95%CI:0.03, 0.28) -Significant positive associations between ΣHMWP and E2 (β:0.22pg/mL; 95%CI:0.01, 0.13)
*Organophosphate pesticides*							
Qin et al. [52]	306 mother-child pairs from the LWBC cohort from Shandong Province, China	Cohort*	6 DAPs: DMP, DMTP, DMDTP, DEP, DETP, and DEDTP measured with GC-MS/MS	Maternal midstream spot urine samples at delivery	E2 and T measured with RIA	Cord serum at delivery	-Inverse associations between DEP and E2 (β:−0.03pg/mL, 95%CI:−0.07, − 0.00); DMP and T (β:−0.08ng/mL, 95% CI: -0.13, -0.03); DETP and T (β:−0.08ng/mL, 95%CI:-0.14, -0.01); DAPS and T (β:−0.10ng/mL, 95%CI:-0.17, -0.03); DMP and T/E2 (β:−0.06, 95%CI: -0.10, -0.01); DETP and T/E2 (β:−0.07, 95%CI:-0.13, -0.01) -In participants carrying males, significant association between DETP and T/E2 (β:-0.09, 95%CI:-0.17, -0.00) -Significant inverse associations in participants carrying females included: DMP (β:−0.05pg/mL, 95%CI: -0.07, -0.02), DEP (β:−0.06pg/mL, 95%CI: -0.09, -0.02), DETP (β:−0.04pg/mL, 95%CI:-0.08, -0.00), and DAPS (β:−0.07pg/mL, 95%CI: -0.11, -0.04) with E2; DMP (β:−0.12ng/mL, 95%CI:-0.18, -0.06), DETP (β:−0.10ng/mL, 95%CI:-0.18, -0.02), and DAPs (β:−0.13ng/mL, 95%CI: -0.21, -0.05) with T; DMP and T/E2 (β:−0.07, 95% CI:-0.13, -0.02) -Similar results when exposures examined categorically

*Abbreviations* BEP = benzylparaben; BPA = bisphenol A; BPAF = bisphenol AF; BPA-g = BPA-glucuronide; BPAP = bisphenol AP; BPB = bisphenol B; BPF = bisphenol F; BPP = bisphenol P; BPS = bisphenol S; BPZ = bisphenol Z; BP-3 = benzophenone-3; BuPB = butylparaben; Cx-MiNP = mono(carboxy-methyl-heptyl) phthalate; DAP = dialkylphosphate; DEDTP = diethyldithiophosphate; DEP = diethylphosphate; DETP = diethylthiophosphate; DMDTP = dimethyldithiophosphate; DMP = dimethylphosphate; DMTP = dimethylthiophosphate; E1 = estrone; E2 = estradiol; E3 = estriol; ELISA = enzyme-linked immunosorbent assay; EPB = ethylparaben; GC-MS/MS = gas chromatography tandem mass spectrometry; HPLC-MS/MS = high-performance liquid chromatography with tandem mass spectrometry; ID-HPLC- ESI-MS/MS = isotope dilution-high performance liquid chromatography-electrospray ionization-tandem mass spectrometry; ID-LC/MS/MSL = isotope dilution-liquid chromatography-tandem mass spectrometry; IDMS = isotope dilution mass spectrometry; LC-MS = liquid chromatography mass spectrometry; LC-ESI-MS/MS = liquid chromatography/electrospray tandem mass spectrometry; LC-MS/MS = liquid chromatography with tandem mass spectrometry; LOD = limit of detection; MBP = mono-butyl phthalate; MBzP = monobenzyl phthalate; MCHP = monocyclohexyl phthalate; MCHPP = mono-(7-carboxy-n-heptyl) phthalate; MCMHP: mono(2-carboxymethylhexyl) phthalate; MCNP = mono(carboxynonyl) phthalate; MCOCH = cyclohexane-1,2-dicarboxylic acid monocarboxy isooctyl ester; MCOMHP = mono (6-COOH-2-methylheptyl) phthalate; MCOP = mono-carboxyisooctyl phthalate; MCPP = mono(3-carboxypropyl) phthalate; MECPP = mono-2-ethyl-5-carboxypentyl phthalate; MECPTP = mono-2-ethyl-5-carboxypentyl terephthalate; MEHP = mono-(2-ethylhexyl) phthalate; MEHHTP = mono-2-ethyl-5-hydrohexyl terephthalate; MEHHP = mono-2-ethyl-5-hydroxyhexyl phthalate; MEOHP = mono(2-ethyl-5-oxohexyl) phthalate; MEP = mono-ethyl phthalate; MHBP = mono-hydroxybutyl phthalate; MHiBP = monohydroxyisobutyl phthalate; MHiNCH = cyclohexane-1,2-dicarboxylic acid monohydroxy isononyl ester; MHpP = mono-2-heptyl phthalate; MHXP = mono-hexyl phthalate; MiBP = mono-isobutyl phthalate; MINP = mono-isononyl phthalate; MIPRP = monoisopropyl phthalate; MMP = monomethyl phthalate; MnPeP = mono-n-pentyl phthalate; MnOP = mono-n-octyl phthalate; MONP = mono oxononyl phthalate; MPB = methylparaben; MPEP = mono-n-pentenyl phthalate; OH-MnBP = mono(hydroxy-n-butyl) phthalate; OH-MiNP = mono(hydroxyl-methyl-octyl) phthalate; ohMPHP = 6-hydroxy-mono-propyl-heptyl phthalate; oxo-MiNP = mono(oxo-methyl-octyl) phthalate; P = progesterone; PPB = propylparaben; RIA = radio-immunoassay; T = testosterone; TCC = Triclocarban; TCS = Triclosan; TQMS = triple quadrupole mass spectrometer; UHPLC = Ultra High-Performance Liquid Chromatography; UPLC-MS/MS = Ultra-performance liquid chromatography-mass spectrometry; 2,4-DCP = 2,4-dichlorophenol; 2,5-DCP = 2,5-dichlorophenol; 4-NP = 4-nonylphenol; 4-tOP = 4-tert-octyphenol; ∑DBP = sum of di-n-butyl phthalate metabolites; ∑DEHP = molar sum of di(2-ethylhexyl) phthalate metabolites; ∑DiBP = molar sum of di-iso-butyl phthalate metabolites; ∑DINCH = molar sum of di(isononyl)cyclohexane-1,2-dicarboxylate; ∑DiNP = molar sum of di-iso-nonyl phthalate metabolites; ∑DnBP = molar sum of di-n-butyl phthalate metabolites; ∑DnOP = molar sum of di-n-octyl phthalate metabolites; ∑HMWP = molar sum of high-molecular-weight phthalate metabolites; ∑LMWP = molar sum of low-molecular-weight phthalate metabolites

*Indicates a pregnancy cohort with at least one cross-sectional analysis

**Table 3:** Summary of epidemiological studies examining the relationship of maternal persistent chemical exposures with maternal and/or fetal sex steroid hormones

First author (year)		Study sample and location	Study design	Exposure measures and assay	Exposure source and timing	Outcome measures and assay	Outcome source and timing		Main findings
*PFAS*									
Rivera-Núñez et al. [19]		285 pregnant people from the UPSIDE cohort in Rochester, NY, USA	Cohort*	PFOA, PFOS, PFNA, PFHxS, and PFDA measured by HPLC-MS/MS	Second trimester maternal serum	E1, E2, E3, T, and fT measured with HPLC-MS/MS	Maternal serum in each trimester		-In longitudinal models, PFHxS associated with higher T (%∆: 19.9, 95%CI:1.7, 41.6); PFNA (%∆:7.9, 95%CI:3.4, 12.5) and PFDA (%∆:7.2, 95%CI: 4.8, 9.7) associated with higher fT -In participants carrying male fetuses: PFHxS (%∆:31.7, 95%CI:1.7, 70.6) and PFNA (%∆:26.3, 95%CI:3.2, 54.2) associated with higher T; PFNA (%∆:8.4, 95%CI:1.4, 15.9) and PFDA (%∆:8.0, 95%CI:5.0, 11.0) associated with higher fT -In participants carrying female fetuses: PFHxS associated with higher E3 (%∆:17.9, 95%CI:3.2, 34.8), PFNA (%∆:7.7, 95%CI:1.7, 14.8) and PFDA (%∆:6.8, 95%CI:2.8, 11.0) associated with higher T -Similar results for trimester-specific models
Yao et al. [28]		369 singleton pregnancies in Shandong, China	Cohort*	PFOA, PFOS, PFNA, PFDA, PFUdA, and PFHxS measured by HPLC-MS/MS	Maternal serum at hospital admission (within 3 days of delivery)	E2 and T measured by RIA	Cord serum at delivery		-PFOA positively associated with E2 (β:0.03 pg/mL, 95%CI:0.00, 0.07) -PFUdA positively associated with T (β:0.13 ng/mL, 95%CI:0.00, 0.26)
Itoh et al. [26]		189 Japanese mother-infant pairs from the Sapporo Cohort	Cohort	PFOS and PFOA measured by LC/MS/MS	Mid-late pregnancy maternal serum	E2, T, and P measured using LC-MS/MS;	Cord blood at delivery		-PFOS positively associated with E2; only significant among those carrying males E2 (β:0.37ng/mL, 95%CI:0.06, 0.69) -PFOS inversely associated with P in those carrying male (β:-0.34ng/mL, 95%CI:-0.68, -0.01) and female fetuses (β:-0.55ng/mL, 95%CI:-0.89, -0.21) -Association between PFOS and T was inverse in those carrying males and positive in those carrying females, but not significant -No significant associations with PFOA
Kobayashi et al. [27]		224 pregnant Japanese people from the Sapporo cohort	Cohort	PFOS and PFOA measured by LC/MS/MS	Late pregnancy or post-delivery maternal serum	P, T, and E2 using LC/MS/MS	Cord blood at delivery		-PFOS associated with lower P in the full cohort (β:-0.40ng/mL, 95%CI:-0.60, -0.20), those carrying male fetuses (β:-0.27ng/mL, 95%CI:-0.51, -0.03), and female fetuses (β:-0.53ng/mL, 95%CI:-0.83, -0.21) -PFOS positively associated with E2 (β:0.17ng/mL, 95%CI:0.01, 0.33) in the full cohort and in both sexes -The association between PFOS and T was inverse in those carrying males and positive in those carrying females, but not significantly so -No significant associations between PFOA and sex hormones
Yang et al. [29]		557 pregnant people in China	Cohort*	PFOA, PFNA, PFHxA, PFDA, PFUdA, PFOS, PFDoA, PFHxS, PFHpS, PFBS, and PFHpA measured by UPLC-Q/ TOF MS	Early pregnancy maternal serum	E1, E2, and E3 measured by ultra-performance LC coupled to quadrupole time-of-flight MS	Maternal serum collected at 3 time points (5–15 weeks, 24–28 weeks, 36–40 weeks)		-In the first trimester PFUdA negatively associated with E1 (β:-0.06ng/mL, 95%CI: -0.10, -0.02) and E3 (β:-0.04ng/mL, 95%CI:-0.07, -0.11); PFOS negatively associated with E1 (β:-0.11ng/mL, 95%CI:-0.22, -0.003) -PFDA negatively associated with mid-pregnancy E1 (β:-0.21ng/mL, 95%CI:-0.36, -0.05) -PFNA negatively associated with E2 in late pregnancy (β:-0.13ng/mL, 95%CI:-0.25, -0.02) -In linear mixed models, PFDA negatively associated with E1 across timepoints (β:-0.12 ng/mL, 95%CI: -0.24, -0.01)
Qin et al. [30]		879 mother-child pairs from Maoming, China (371 preterm birth cases and 508 controls)	Nested case-control	PFBA, PFHxS, PFOA, PFOS, and PFNA measured with UPLC-MS/MS	Maternal serum (mean gestational age: 32 weeks, range: 7–40 weeks)	E2 and E3 measured by immunoluminometric assay	Cord serum at delivery		-Br-PFHxS weakly associated with E2 (β: 0.03 ng/mL, 95%CI: 0.01, 0.06) - PFNA, PFOA, PFHxS also had positive, non-significant associations with E2 -Inverse associations between E2 with PFOS (β:-0.04ng/mL, 95%CI:-0.08, -0.01), PFBA (β:-0.02ng/mL, 95%CI: -0.03, -0.00), and a nonsignificant inverse association with Br_PFOS (β:-0.03ng/mL, 95%CI:-0.08, -0.01) -All PFAS (but PFBA) non-significantly positively associated with E3
Bonefeld-Jorgensen et al. [31]		800 nulliparous people with singleton pregnancies from the FETOTOX project in Denmark	Biobank Cohort*	PFOS, PFHxS, PFHpS, PFOA, PFDA, PFNA, PFUnA, and PFHpA -measured with solid-phase extraction and LC-MS/MS	Maternal serum < 14 weeks gestation	E1 and E2 measured by tandem mass spectrometry	Maternal serum < 14 weeks gestation		-Compared to those in Q1 of PFDA, Q2 (β:1467pmol/l, 95%CI:400.0, 2533), Q3 (β:1464pmol/l, 95%CI:380.9, 2547), and Q4 (β:1384pmol/l, 95%CI:282.7, 2486) had higher E2; those in Q3 (β:781.2pmol/l, 95%CI:144.1, 1418) had higher E1 -Compared to those in Q1 PFOA, those in Q2 (β:-1424pmol/l, 95%CI:-2509, -358.7) had lower E2 and those in Q4 (β:-764.3pmol/l, 95%CI:-1396, -132.4) had lower E1 -No other PFAS associated with estrogens
Nian et al. [32]		752 mother-infant pairs from the Shanghai birth cohort	Cohort	PFOA, PFOS, PFNA, PFDA, PFUnDA, PFHxS, PFDoA, PFBS, and PFHpA measured with HPLC-MS/MS	Maternal plasma (1st to 3rd quartile: 13–17 weeks; median: 15 weeks)	T measured using chemiluminescence immunoassay	Cord serum at delivery		-PFHxS significantly associated with higher T (β:0.06nmol/L, 95%CI:0.003, 0.12) in the full cohort -Significant nonlinear association between PFUnDA and TT (Pnonlinear = 0.02) in restricted cubic spline analyses
*Polychlorinated biphenyls*									
Bonefeld-Jorgensen et al. [31]		197 pregnant people from the FETOTOX project in Denmark	Cohort biobank*	Seven PCB congeners (PCB138, − 153, −180, − 170, −118, − 187 and − 156) and ΣPCB measured with LC-MS/MS	First trimester maternal serum	E1 and E2 measured via MS/MS	First trimester maternal serum		All PCBs non-significantly, inversely associated with E1 and E2
Cao et al. [49]		104 mother-infant pairs in Duisburg, Germany	Cohort*	Σnon-o-PCB, Σmono-o-PCB, and six indicator PCBs (congener 28, 52, 101, 138, 153, and 180) used for Σ6 PCB measured with HRGC/HRMS	Late pregnancy maternal whole blood	T and E2 measured with chemiluminescence immunoassay	Late pregnancy maternal and cord serum at delivery		-In the full cohort Σnon-o- PCB associated with T (means ratio:0.85, 95%CI:0.75, 0.96) and Σmono-o-PCB and T (means ratio:0.85, 95% CI:0.73, 1.01) -In the full cohort, Σ non-o- PCB (means ratio:0.83, 95%CI:0.73, 0.95), Σ mono-o-PCB, (means ratio:0.81, 95%CI:0.68, 0.97), and Σ_6_ PCB (means ratio:0.85, 95% CI:0.72, 1.01) inversely associated with E2 -Among those carrying female fetuses, Σ non-o- PCB (means ratio:0.81, 95%CI: 0.69, 0.94), Σmono-o-PCB, (means ratio: 0.72, 95%CI:0.57, 0.90), and Σ_6_ PCB (means ratio:0.76, 95%CI:0.61, 0.96) associated with lower T, while those carrying males had non-significantly higher T -In males, Σ non-o- PCB was associated with E2 (means ratio:0.76, 95% CI:0.58, 0.99). Those carrying females also had a non-significant inverse association (means ratio:0.90, 95% CI:0.78, 1.04)
Miyashita et al. [50]		183 mother-child pairs from the Sapporo Cohort in Japan	Cohort*	4 non-ortho PCBs, and 8 mono-ortho PCBs measured with HRGC/HRMS	Maternal whole blood in second or third trimester, or right after delivery	E2, T, and P measured with LC-MS/MS	Cord blood at delivery		-No associations between Sub-total non-ortho PCBs or Sub-total mono-ortho PCBs with P, E2, T, or T/E2 in full sample -In males, non-*ortho* PCBs associated with lower T/E2 ratio in mothers carrying males (β:-0.22, 95%CI: -0.42, -0.03). Non-significant inverse associations in mothers carrying females -Significant interaction terms between sex and Sub-total non-ortho PCBs and T/E2 ratio (*p* = 0.018) and sex and total DLCs and T/E2 (0.049)
*Polybrominated diphenyl ethers*									
Gao et al. [51]	125 pregnant people from the LWBC Cohort in Shandong province, China		Cohort*	PBDE congeners: BDE-28, -47, -85, -99, -100, -153, -154, and − 183; ∑5PBDEs created with BDE-28, -47, -99, -100, and − 153 measured with GC-MS/MS	Maternal serum at admission for labor (term)	E2 and T measured with RIA	Maternal serum at admission for labor (term)	No associations between hormones and PBDE concentrations	
*Organochlorine pesticides*									
Araki et al. [17]	232 mother-child pairs from the Sapporo Cohort in Japan		Cohort*	cis-chlordane, trans-chlordane, cis-nonachlor, trans-nonachlor, oxychlordane, 6 DDTs (o, p′-DDT, p,p′-DDT, o,p′-DDE, p,p′-DDE, o,p′-DDD, and p, p′-DDD), aldrin, dieldrin, endrin, heptachlors, cis-HCE, trans-HCE, HCB, 4 HCH isomers (α-HCH, β-HCH, γ-HCH, and δ-HCH), Mirex, and 6 toxaphenes (Parlar-26, Parlar-41, Parlar-40, Parlar-44, Parlar-50, and Parlar-62) measured using GC-MS/MS and CG-NICI-MS	Maternal serum at recruitment or within a week after delivery	P, E2, and T measured with LC-MS/MS	Cord serum at delivery	-There was a decreasing trend for T in relation to quartiles of Mirex (p = 0.039), while E2/T had an increasing trend in relation to p, p’-DDE quartiles (*p* = 0.02) -Among participants carrying males, Mirex associated with lower T (β:-0.26pg/mL, 95%CI: -0.49, -0.32) and p, p’-DDE associated with higher E2/T (β:0.30, 95%CI: 0.05, 0.54) -No associations between OCPs and P or E2 for either sex	
Bonefeld-Jorgensen et al. [31]	197 pregnant people from the FETOTOX project in Denmark		Cohort biobank*	∑11 OCPs (∑OCP): HCB, p, p′-DDE, HCH, oxychlordane, and transnonachlor measured with LC-MS/MS	First trimester maternal serum	E1 and E2 measured with MS/MS	First trimester maternal serum	-In the total cohort HCB associated with E2 (β:-264pmol/l.2, 95%CI:-445.2, -83.2) and E1 (β:-147.0pmol/l, 95% CI:-286.4, -7.5). No other significant associations, but most were inverse -Results similar when exposures were examined categorically	
**Dioxins**									
Cao et al. [49]		104 mother–infant pairs in Duisburg, Germany	Cohort	∑ of 17 PCDD/F congeners measured with HRGC/HRMS	Late pregnancy maternal whole blood	T and E2 measured using chemiluminescence immunoassay	Cord serum at delivery		-PCDD/F WHO-TEq-weighted ∑associated with T among those carrying female fetuses (means ratio:0.69, 95%CI:0.53–0.90), no associations in male fetuses and marginally significant in the full cohort (means ratio:0.84, 95%CI:0.71–1.01) - PCDD/F WHO-TEq-weighted ∑associated with E2 in the total cohort (means ratio:0.73, 95%CI:0.60–0.87), those carrying male fetuses (means ratio:0.74, 95%CI:0.55–1.00), and female fetuses (means ratio:0.75, 95%CI:0.60–0.94)
Miyashita et al. [50]		183 mother-child pairs from the Sapporo Cohort in Japan	Cohort*	7 PCDDs, 10 PCDFs, total dioxin-like-compound composed of 7 PCDDs, and 10 PCDFs measured with HRGC/HRMS	Maternal whole blood samples collected during pregnancy or at delivery	E2, T, and P measured using LC-MS/MS	Cord blood at delivery		-No associations between sub-total PCDDs and sub-total PCDFs with P, E2, T, or T/E2 in the full cohort. Associations trended inverse for P and T/E2 and positive for E2 -When stratified, associations with sub-total PCDDs or Sub-total PCDFs and T/E2 were not significant, but were inverse among those carrying males and positive for those carrying females

*Abbreviations* br-PFHxS = perfluorohexanesulfonic acid and its branched isomer; br-PFOS = perfluorooctanesulfonic acid and its branched isomer; CG-NICI-MS = gas chromatography/negative-ion chemical-ionization mass spectrometry; DDD = dichlorodiphenyldichloroethane; DDE = dichlorodiphenyldichloroethylene; DDT = dichlorodiphenyltrichloroethane; E1 = estrone; E2 = estradiol; E3 = estriol; fT = free testosterone; GC-MS/MS = gas chromatography tandem mass spectrometry; HCB = hexachlorobenzene; HCH = hexachlorocyclohexane; HPLC–MS/MS = high-performance liquid chromatography coupled with tandem mass spectrometry; HRGC/HRMS = high-resolution gas chromatography/high-resolution mass spectrometry; LC-MS/MS = liquid chromatography–tandem mass spectrometry; MS/MS = Tandem mass spectrometry; OCP = organochlorine pesticides; P = progesterone; PBDE = polybrominated diphenyl ether; PCB = polychlorinated biphenyls; PCDD = polychlorinated dibenzo-p-dioxins; PCDD/F = polychlorinated dibenzo-p-dioxins and dibenzofuran; PCDF = polychlorinated dibenzofuran; PFBA = perfluorobutanoic acid; PFBS = perfluorobutanesulfonate; PFDA = perfluorodecanoic acid; PFDoA = perfluorododecanoic acid; PFHpA = perfluoroheptanoic acid; PFHpS = perfluoroheptane sulfonate; PFHxA = perfluorohexanoic acid; PFHxS = perfluorohexane sulfonic acid; PFNA = perfluorononanoic acid; PFOA = perfluorooctanoic acid; PFOS = perfluorooctanesulfonic acid; PFUdA = perfluoroundecanoic acid ; PFUnDA = perfluoronundecanoic acid; RIA = radio-immunoassay; T = testosterone; Trans-HCE = trans-heptachlor epoxide; UPLC-MS/MS = ultra-performance liquid chromatography–tandem mass spectrometry; UPLC-Q/TOF MS = ultra-performance liquid chromatography coupled to quadrupole time-of-flight mass spectrometry; ∑mono-o-PCB = sum of eight mono ortho polychlorinated biphenyl congeners, ∑non-o-PCB = four non ortho polychlorinated biphenyl congeners

*Indicates a pregnancy cohort with at least one cross-sectional analysis

**Table 4:** Overall trends of associations between maternal exposure to synthetic chemicals (by class) and sex steroid hormones

	Androgens		Estrogens		Progesterone	
	No. of papers	Direction of assoc.	No. of papers	Direction of assoc.	No. of papers	Direction of assoc.
Phenols	6	↓	8	↓	4	↓
Parabens	3	↓	4	↓	3	↓
Phthalates	7	↑↓	8	↑↓	4	↓
PFAS	5	↑↓	7	↑↓	2	↓
PCBs	2	↓	3	↓	1	-
PBDEs	1	-	1	-	0	N/A
Dioxins	2	↓	2	↑↓	1	↓
Organochlorine pesticides	1	↓	2	↑↓	1	-
Organophosphate pesticides	1	↓	1	↓	0	N/A

*Abbreviations* PFAS = poly- and perfluoroalkyl substances; PCBs = polychlorinated biphenyl; PBDEs = polybrominated diphenyl ethers

↓ = Associations were generally inverse

↑↓ = Associations were generally mixed

- = Null

## Results

### Summary of Search Results

**Fig. 1:**
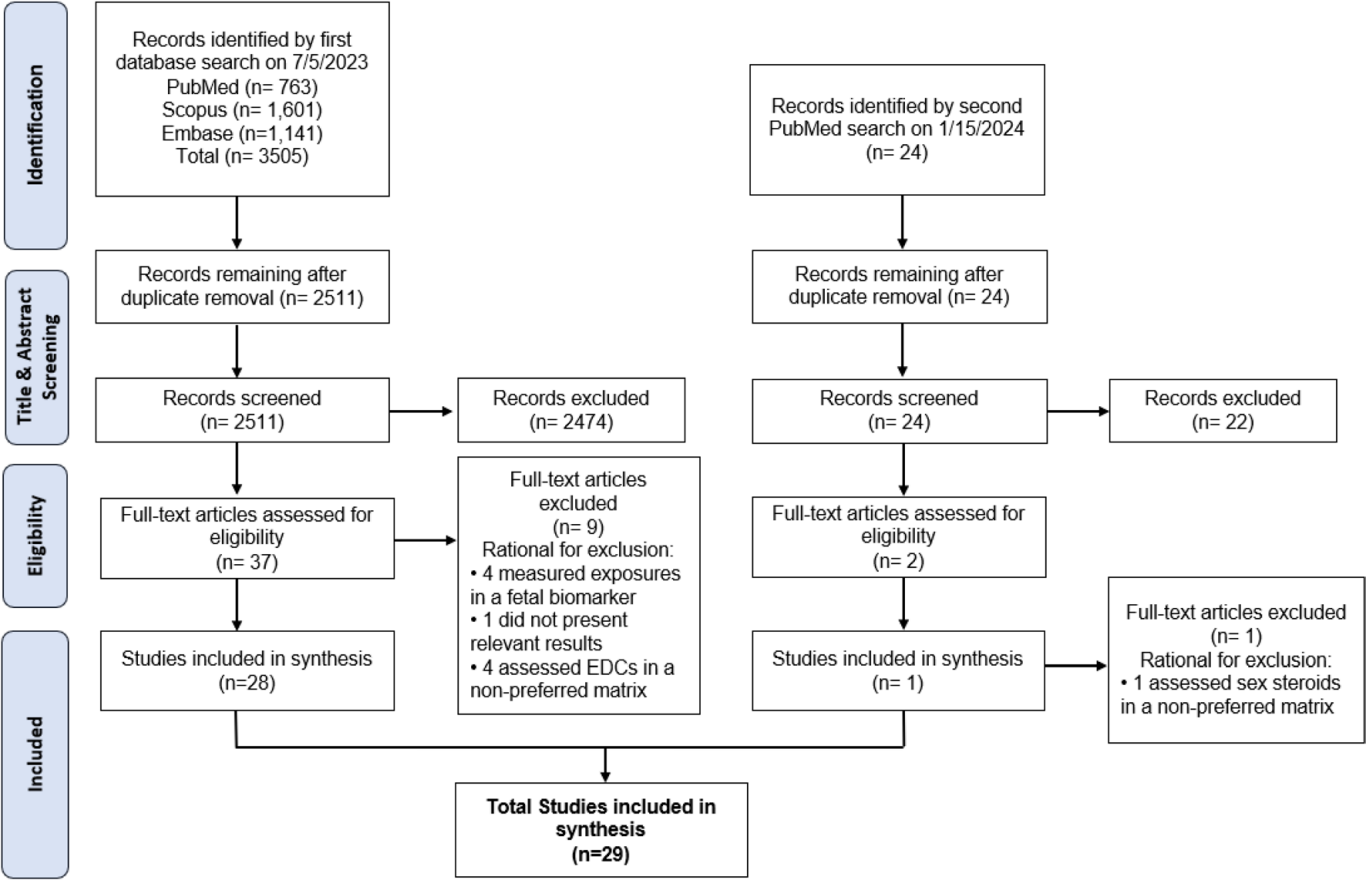
Flow chart of study selection

Searches across the three databases yielded a total of 3,505 articles. After duplicate removal, 2,511 papers remained for screening. The title and abstract screening process resulted in the exclusion of 2,474 articles, with 37 moving forward to full-text review. After full-text review, 28 articles were selected for narrative review. The search rerun resulted in the identification of 24 new papers. Following screening, one paper met the inclusion criteria, resulting in a final count of 29 papers to be reviewed (Fig. 1). Five studies were excluded for using a non-preferred maternal matrix. These studies measured phenols and parabens in plasma or serum, phthalates in plasma, and sex steroids in hair [21–25]. There were 9 articles on phenols, 9 on phthalates, 8 on PFAS, 4 on parabens, 3 on PCBs, 3 on pesticides, 2 on dioxins, and 1 on flame retardants. The included studies represent 19 pregnancy cohorts from geographically diverse areas including the United States, China, South Africa, and Germany. Study sizes varied from 56 participants to 879 participants. Across studies, median concentrations of chemicals varied for some compounds, but were relatively similar for others. For example, median perfluorooctanoic acid (PFOA) ranged from 0.59-42.83ng/mL, while median Bisphenol A (BPA) ranged from 0.68-4.0ng/mL [19, 26–32].

### Parabens & Phenols

Nine studies investigated paraben and phenol concentrations in relation to maternal and/or fetal sex steroid hormones (Table 2).

*Progesterone.* Preliminary analyses from the PROTECT cohort, which recruited pregnant people in Puerto Rico (*n* = 106), showed non-significant inverse associations between phenols and parabens (methylparaben [MPB] and propylparaben [PPB]) and phenols (BPA, triclosan [TCS], benzophenone-3 [BP-3], 2,4-dichlorophenol [2,4-DCP], and 2,5-dichlorophenol [2,5-DCP]) and progesterone at multiple timepoints across pregnancy [33]. A larger PROTECT study expanded upon this preliminary analysis (*n* = 602) and similarly observed no significant associations in mixed models [34]. By contrast, a smaller cohort from Michigan (MMIP; *n* = 56) reported a positive association between butylparaben (BuPB) and cord plasma progesterone (β:0.35nmol/L, Benjamini-Hochberg false discovery rate (BH-FDR):0.096; a BH-FDR of 0.1 was considered significant) [35]. Finally, a New York cohort (*n* = 139) reported a non-significant inverse association between ΣBisphenols and progesterone (%Δ:-4.8; 95%CI:-10.4, 1.1) [36].

*Androgens.* In the full PROTECT cohort (*n* = 602), BPA was associated with 17% lower T (95%CI:-26.7, -6.87) at 24–28 weeks gestation [34]. Likewise, a Chinese cohort (*n* = 137) reported that a doubling of BPA was associated with lower T (β:-31.1ng/dl; 95%CI:-53.07, -9.11) and compared to those in the lowest quartile, those in the highest BPA quartile had significantly lower T (β:−180ng/dl; 95%CI:-333.5, -26.2) [37]. Three studies observed associations between TCS and T. In a second Chinese cohort (*n* = 537), compared to those below the limit of detection for TCS, participants with the highest concentrations had higher cord serum T (β:0.09ng/mL; 95%CI:0.02, 0.16), results that were stronger in males than females [38]. However, Guo et al. (2021) observed an inverse association between TCS and cord serum T, which was significant in females (β:-0.08ng/mL; 95%CI:-0.14, -0.01) [39]. In the MMIP cohort, sex differences in the association between ethylparaben (EPB) and maternal T at term were noted, specifically a non-significant, positive association in participants carrying males (β:0.09nmol/L, BH-FDR:0.69), and a stronger, inverse association in those carrying females (β:-0.25nmol/L, BH-FDR:0.02) [35].

*Estrone (E1)*: In 851 Chinese pregnancies, in each trimester, participants in the highest BPA quartile had lower E1 compared to those in the lowest quartile, with some differences noted by trimester and fetal sex [40].

*Estradiol (E2)*: Multiple studies reported inverse associations between parabens and phenols and E2. Li et al. (2020) found that compared to the lowest BPA quartile, those in the higher quartiles had lower E2 in the first (Q2β:-0.23ng/mL, 95%CI:-0.37, -0.09; Q3β:-0.21ng/mL, 95%CI:-0.35, -0.07), second (Q2β:-0.19ng/mL; 95%CI:-0.34, -0.04), and third trimester (Q3β:-0.17ng/mL; 95%CI:-0.32, -0.02), with some stronger associations observed in males [40]. A second study also observed inverse associations between BPA and cord serum E2 in both sexes that were stronger among participants carrying males (β:-0.05pg/mL; 95%CI:-0.11, -0.001) [39], while others observed no associations between BPA and E2 [33, 37, 39]. Associations between TCS and E2 have conflicted across studies with some studies showing inverse associations [39, 41], while others showed opposing directionality depending on fetal sex [38]. Although some larger studies reported null results [34, 39], a smaller study (*n* = 56) reported a negative association between BP-3 and first trimester E2 (*P* < 0.05) [35]. For parabens, in the PROTECT cohort, an interquartile range (IQR) increase in BuPB was associated with 8.46% lower maternal E2 (95%CI:-16.92, 0.00) and there were negative associations between MPB and PPB with E2 at weeks 16–20, but positive associations at weeks 24–28 [33]. By contrast, no associations between parabens and cord serum E2 were reported in a large Chinese study [39].

*Estriol (E3)*: Li et al. (2020) reported non-monotonic associations between phenols and E3. In each trimester, compared to those in the lowest BPA quartile, those in quartiles 2 and 3 had lower E3, while those in the highest quartile (Q4) had higher E3 (second trimester β:0.20ng/mL; 95%CI:0.02, 0.38) [40]. Banker et al. [35] reported that negative associations between E3 and BPA and 2,4-DCP were strongest in participants with a high BMI carrying female fetuses [35]. Lastly, no associations between phenols and E3 were observed in PROTECT [34].

### Phthalates

To our knowledge, to date, nine publications have reported on maternal urinary phthalate exposure in relation to maternal or cord sex steroids (Table 2).

*Progesterone.* Using linear mixed models, in PROTECT (*n* = 106), an IQR increase in mono-ethyl phthalate (MEP) was associated with 10.6% lower maternal progesterone (95%CI:-17.6, -2.84), with stronger associations in early versus late gestation [42]. In a larger PROTECT analysis (*n* = 677), inverse associations with progesterone were again observed, most notably with mono-carboxyisooctyl phthalate (MCOP) in early gestation (Δ%:-10.8, 95%CI:-19.4, -1.2) and mono-2-ethyl-5-hydrohexyl terephthalate (MEHHTP) in late gestation (Δ%:-13.1, 95%CI:-22.3, -2.8) [18]. In a New York cohort (*n* = 139), higher early-mid pregnancy ∑di-n-octyl phthalate (ΣDnOP) (Δ%:-8.1, 95%CI:-15.2, -0.4) and ∑di-iso-nonyl phthalate metabolites (ΣDiNP) (Δ%:-7.7, 95%CI:-13.3, -1.7) were similarly associated with lower second trimester progesterone [36]. Lastly in the MMIP cohort (*n* = 56), no associations were observed [35].

*Androgens.* In the multi-center U.S. TIDES cohort (*n* = 591), early pregnancy mono(carboxynonyl) phthalate (MCNP) (∆%:-12.40, 95%CI:-22.48, -1.03) and mono-2-ethyl-5-carboxypentyl phthalate (MECPP) (∆%:-12.30, 95%CI:-21.17, -2.43) were inversely associated with free T, with weaker inverse trends noted for additional di(2-ethylhexyl) phthalate (DEHP) metabolites [43]. A different set of metabolites of concern emerged in the PROTECT cohort, where an IQR increase in MEP was associated with lower T (∆%:-14.5, 95%CI:-24.3, -3.42), while an IQR increase in mono-hydroxybutyl phthalate (MHBP) was associated with higher T (∆%:4.71, 95%CI:0.27, 9.35) [18]. In the I-KIDS study (Illinois, U.S., *n* = 439), few associations were observed between phthalate metabolites (measured in urine samples pooled across pregnancy) and a summary measure of urinary androgens [44]. Reports of sex differences have been inconsistent. For example, in the U.S. Study for Future Families (SFFI; *n* = 180), mid-late pregnancy mono-butyl phthalate (MBP) and ΣDEHP were associated with lower total T in mothers carrying males, whereas MEP was associated with higher total T in mothers carrying females [45]. Lin et al. [46] also noted differences by fetal sex, whereby multiple third trimester phthalate metabolites (MEP, mono-(2-ethylhexyl) phthalate [MEHP], and mono-2-ethyl-5-hydroxyhexyl phthalate [MEHHP]) were inversely correlated with cord serum free T in mothers carrying females, but not males [46]. Overall, this literature shows conflicting results, while a broader literature shows that phthalates may act on androgen pathways through multiple mechanisms [47].

*Estrogens.* Several studies reported associations between phthalates and estrogens in pregnancy. In TIDES, first trimester urinary mono-isobutyl phthalate (MiBP) was associated with higher E1 (∆%:30.38, 95%CI:9.02, 55.96) and E2 (∆%:29.99: 95%CI:12.20, 50.59), as were mono(2-ethyl-5-oxohexyl) phthalate (MEOHP) and MEHP [43]. Monobenzyl phthalate (MBzP) was additionally associated with higher E2 (∆%:14.66, 95%CI:1.30, 29.75) [43]. In a pregnancy cohort from Slovakia (*n* = 90), Hlisnikova et al. (2022) also found positive associations between early pregnancy mono(hydroxyl-methyl-octyl) phthalate (OH-MiNP) (β:0.24pg/mL; 95%CI:0.03, 0.28) and the ∑of high-molecular-weight phthalate metabolites (ΣHMWP) (β:0.22pg/mL; 95%CI:0.01, 0.13) with E2 [48]. Fewer studies have examined associations with E3, with Cathey et al. (2019) reporting null associations and Banker et al. (2021) reporting inverse associations with MEHP, but only among mothers with higher BMI carrying female fetuses [18, 35]. In an analysis of ∑urinary estrogen metabolite concentrations across pregnancy, associations with phthalate metabolites and substitutes tended to be positive [44]. Lastly, several studies examined maternal phthalate exposure in relation to hormone ratios. For example, in a Taiwanese cohort (*n* = 155), among people carrying female fetuses, higher third trimester ΣDEHP was associated with reduced free T: E2 [46]. Other studies have reported conflicting results, however, with lower ratios of urinary androgen to estrogen metabolites among participants with higher exposure to ΣDEHP, mono(3-carboxypropyl) phthalate (MCPP), and MEP [44].

### PFAS

Eight publications have examined gestational PFAS in relation to sex steroid hormones, with mixed results (Table 3).

*Estrogens.* Seven studies examined serum PFAS concentrations in relation to maternal or cord estrogens. In longitudinal analyses in the U.S. UPSIDE cohort (*n* = 285), perfluorohexane sulfonic acid (PFHxS) was associated with higher estrogens in participants carrying female, but not male, fetuses (%∆E2:13.2, 95%CI:-0.1, 29.1; %∆E3:17.9, 95%CI:3.2, 34.8) [19]. In the Chinese LWBC study (*n* = 351), PFOA was positively associated with E2 (β:0.03pg/mL, 95%CI:0.00, 0.07) [28]. Similarly, in analyses based on the Hokkaido study, Itoh et al. (*n* = 189) reported positive associations between perfluorooctanesulfonic acid (PFOS) and cord blood E2 (β:0.37ng/mL, 95%CI:0.06, 0.69), as did Kobayashi et al. (*n* = 224) (β:0.17ng/mL, 95%CI:0.01, 0.33) [26, 27]. In contrast, Yang and colleagues reported negative associations between several PFAS and estrogens in a pregnant population from Hebei Province, China (*n* = 557) [29]. Higher maternal PFOS, perfluorodecanoic acid (PFDA), and perfluoroundecanoic acid (PFUdA) were all associated with significantly lower early pregnancy E1, while perfluorononanoic acid (PFNA) (β:-0.13ng/mL, 95%CI:-0.25, -0.02) was associated with lower late pregnancy E2. In mother-child pairs from Maoming, China (*n* = 879), Qin et al. (2023) reported mixed results, with PFHxS (β:0.03ng/mL, 95%CI: 0.01, 0.06) being positively associated with E2, while PFOS (β:-0.04ng/mL, 95%CI:-0.08, -0.01) and perfluorobutanoic acid (PFBA) (β:-0.02ng/mL, 95%CI: -0.03, -0.00) showed inverse associations [30]. All PFAS were positively, but non-significantly, associated with E3. Lastly, Bonefeld-Jorgensen et al. [31] reported null associations for continuous PFAS and estrogens in Danish pregnant people (*n* = 800), though some mixed associations were noted by quartile [31].

*Androgens.* Three of five studies on PFAS and androgens have reported positive associations. Rivera-Nunez [19] observed that PFHxS was associated with higher TT (%∆:20.0, 95%CI:1.7, 41.6), an association that was stronger as pregnancy progressed, particularly in those carrying male fetuses [19]. Additionally, PFNA (%∆:7.9, 95%CI:3.4, 12.5) and PFDA (%∆:7.2, 95%CI:4.9, 9.7) were associated with higher TT across pregnancy. Nian [32] also reported higher TT levels with a one ln-unit increase in PFHxS (β:0.06nmol/L, 95%CI:0.003, 0.12), but observed no significant associations after stratifying by fetal sex [32]. Yao (2021) also found positive associations between PFAS and cord serum T, with a significant association between PFUdA and T (β:0.14ng/mL, 95%CI:0.00, 0.27) [28]. However, both Itoh [26] and Kobayashi [27] had null findings with PFOS and PFOA [26, 27].

*Progesterone*: Two Hokkaido study-based analyses examined second trimester PFAS in relation to progesterone. Kobayashi and colleagues reported negative associations between PFOS and cord blood progesterone in the full sample (β:-0.40ng/mL, 95%CI:-0.60, -0.20), and in both sexes [27]. Itoh et al. [26] also observed negative associations between PFOS and progesterone in the cord blood of both sexes (Male β:-0.34ng/mL; 95%CI:-0.68,-0.01; Female β:-0.55ng/mL; 95%CI:-0.89,-0.21) [26].

### Other Synthetic Chemicals

*Polychlorinated Biphenyl (PCBs) and Flame Retardants (Polybrominated Diphenyl Ethers [PBDEs]).* Four studies have examined associations between PCBs and steroid hormones (Table 3). Within the Aarhus Birth Cohort Biobank, amongst participants selected for the FETOTOX project (*n* = 1533), 197 participants contributed data on PCBs and sex steroid hormones [31]. Fourteen PCBs (PCB 28, 52, 99, 101, 105, 118, 128, 138, 153, 156, 170, 180, 183, 187), E1, and E2 were examined in first trimester maternal blood. Overall, non-significant, inverse associations between continuous ΣPCB, E1 and E2 were observed. Participants in the highest ∑PCB quartile had significantly lower E2 than those in the lowest ∑PCB quartile (β:-1964pmol/l, 95%CI:-3786, -142). Models considering individual PCBs also trended in the negative direction, with the strongest result for PCB118 in relation to E2 (β:-245.9pmol/l, 95%CI:-527.0, 36.1).

In the German Duisburg cohort, late pregnancy PCBs, categorized as non ortho (non-o-PCBs), mono ortho PCBs (mono-o-PCB), and Σ_6_PCB (PCB 28, 52, 101, 138, 153, 180) were studied in relation to cord serum E2 and T [49]. Amongst all births, non-o-PCBs were inversely associated with T (means ratio:0.85, 95%CI:0.75, 0.96) and E2 (means ratio:0.83, 95%CI:0.73, 0.95). Results were similar but weaker for mono-o-PCB exposure. In sex stratified analyses, mono-o-PCB and Σ_6_PCB were inversely related to cord serum T in female, but not male, infants and inversely related to E2 in both sexes. These results differ from the Hokkaido Study, where in general, positive trends between PCBs and E2 were observed, while results were null for T [50]. That study also reported sex-specific associations including that non-*ortho* PCBs (Q4 vs. Q1) were significantly associated with lower T/E2 (*p* = 0.007) among males only.

In the only study examining PBDEs, 8 PBDE congeners (BDE-28, -47, -85, -99, -100, -153, -154, and − 183) were examined individually and as a sum (Σ_5_PBDE: BDE-28, -47, -99, -100, and − 153) in pregnant people at delivery [51]. Participants (*n* = 125) were recruited from a high PBDE exposure area in China, however no associations with maternal E2 or T concentrations were observed.

*Pesticides.* Three studies examined prenatal pesticide exposure in relation to sex steroids (Tables 3 and 4). In the Laizhou Wan Birth Cohort (n = 306), numerous non-specific urinary pesticide metabolites were inversely associated with cord serum T, E2, and T/E2 [52]. Results were largely driven by associations in female infants, including associations between dimethylphosphate (DMP) and E2 (β:-0.05pg/mL, 95%CI:-0.07, -0.02), diethylphosphate (DEP) and E2 (β:-0.06pg/mL, 95%CI:-0.09, -0.02), and DMP and T (β:-0.12ng/mL, 95%CI:-0.18, -0.06). Few associations were observed in males save an inverse association between (diethylthiophosphate) DETP and T/E2 ratio (β:-0.09, 95%CI:-0.17, 0.00). Organochlorine pesticides ([OCPS] including 5 chlordanes, 6 dichlorodiphenyltrichloroethanes [DDTs], 3 heptachlors, 3 ‘drins’, 4 hexachlorocyclohexane [HCH] isomers, Mirex, and 6 toxaphenes) and sex steroids were assessed in the Hokkaido cohort [17]. Among male infants, Mirex was associated with lower T (β:-0.26ng/mL, 95%CI:-0.49, -0.32) and dichlorodiphenyldichloroethylene (p, p’-DDE) was associated with higher E2/T (β:0.30, 95%CI:0.05, 0.54); no associations with progesterone or E2 were observed. In contrast to the null findings in the Hokkaido cohort, amongst FETOTOX participants, hexachlorbenzen (HCB) was associated with lower first trimester E2 (β:-264.2pmol/l, 95%CI:-445.2, -83.2) and E1 (β:-147.0pmol/l, 95% CI:-286.4, -7.5); no additional associations between OCPs and hormones were evident [31].

*Dioxins*. Results of the two studies on dioxins and maternal and fetal sex steroid hormones are inconsistent (Table 3). Seventeen maternal polychlorinated dibenzo-p-dioxins and dibenzofurans (PCDD/F) congeners were examined in relation to cord serum T and E2 in Duisberg cohort participants (*n* = 104) [49]. Although no associations with T were observed in the full cohort, amongst female infants, PCDD/Fs were inversely associated with cord serum T (means ratio:0.69, 95%CI:0.53, 0.90). E2 was inversely related to PCDD/Fs amongst the whole cohort (means ratio:0.73, 95%CI:0.60, 0.87) and in both sexes, individually. The Sapporo Cohort of Hokkaido Study examined maternal dioxin-like compounds (7 PCDDs, and 10 PCDFs) from mid and late pregnancy in 183 mother-child pairs in relation to cord blood hormones, reporting null results overall [50]. In models stratified by fetal sex, in males, beta estimates trended negative while in females they trended positive, suggesting sex steroid disruption by dioxins may occur in a fetal sex dependent manner.

## Discussion

In this scoping review, we examined maternal exposure to synthetic chemicals in relation to maternal and fetal sex steroid hormones. The vast majority of studies considered maternal exposure to phthalates and phenols, while other chemicals with endocrine-disrupting potential such as dioxins and pesticides have been considered less often in relation to sex steroids in the epidemiological literature from the examined time period. Generally, studies examining maternal exposure to phenols and parabens showed inverse associations with progesterone, testosterone, and estrogens, with some inconsistencies when stratified by fetal sex (Table 4). Most studies assessing exposure to phthalates and PFAS in relation to progesterone similarly suggested inverse associations, whereas there was less consistency regarding the direction and magnitude of the associations with androgens and estrogens. Lastly, within the remaining chemical classes, in relation to androgens and estrogens, studies examining PCBs generally found inverse associations, studies of pesticide exposure typically found null or inverse associations, and the two dioxin studies observed inverse associations, with some conflicting results when examining by fetal sex.

One theoretical explanation for inconsistent associations across studies is temporal trends in chemical exposure, however the majority of studies recruited participants within the same years (2010–2017). A few older cohorts recruited earlier (from 1999 to 2005), and although one might expect the older cohorts to have higher levels of chemicals like PFAS, as PFAS levels have fallen over the past decades, no notable temporal trends were observed. For example, the Hokkaido Study recruited between 2002 and 2005 and had median PFOS and PFOA concentrations of 5.0ng/mL and 1.4ng/mL, respectively [26, 27]. The remaining cohorts recruited from 2010 to 2019 and had median PFOS and PFOA ranging from 2.5-9.17ng/mL and 0.59-42.83ng/mL, respectively, suggesting some studies were conducted in highly exposed populations, while others are likely more representative of the general population [19, 28–32]. The lack of temporal trend may be in part due to geographical and sociodemographic differences across the cohorts.

Several studies across chemical classes showed that maternal synthetic EDC exposure was inversely associated with progesterone. The epidemiological literature suggests that low levels of progesterone, particularly early in pregnancy, are associated with increased risk of hypertensive disorders of pregnancy and reduced birthweight [53–55]. Phenols, parabens, PCBS, and pesticides were generally associated with lower androgen and estrogen concentrations. Some research suggests that alterations in androgens and estrogens concentrations are associated with pregnancy and child outcomes including low birth weight, preterm birth, and child behavior, however these studies tend to observe adverse outcomes in association with higher steroid concentrations [56, 57]. More research is needed to understand the impact of lower maternal androgen and estrogen concentrations on pregnancy and fetal health. However, there is some suggestion that low levels of estrogens are associated with preeclampsia [58, 59].

The current body of literature on maternal synthetic chemical exposure and sex steroid hormones has several notable strengths. First, while some samples were very small (*n* < 50), most were considerably larger, which is important given the need to additionally examine moderation by fetal sex. Overall, many pregnancy cohorts have pursued questions regarding EDCs and sex steroid hormones, resulting in geographically diverse samples from the United States and Puerto Rico in addition to international samples from countries including China, Denmark, France, Germany, Japan, Slovakia, South Africa, and Taiwan. Limitations and gaps are also noted. Few studies employed mixtures analyses, which more accurately reflect the real-life simultaneous exposure to multiple chemicals. Additionally, establishing consistency across studies is challenging given the many variable factors including the timing of chemical and hormone assessment, examination of sex differences, metabolites and hormones measured, assays used, and modeling approaches. We encourage repeat measures of EDCs and sex steroids in each trimester, which will enable the examination of trimester-specific associations and associations across pregnancy. This may aid in the identification of critical windows of exposure. Further, consistent examination of sex differences is important, as there is evidence that many environmental chemicals impact male and female fetuses differently [19, 35, 46]. Some hormones have received less attention thus far. For example, free testosterone was examined infrequently despite its importance as the biologically active fraction of testosterone [60]. Lastly, select studies examined correlations and these unadjusted results should be viewed with caution. There are also limitations to our scoping review process. A critical assessment of the papers was not performed, and therefore the potential biases of each paper was not considered. Further, our search was limited to studies published from January 1st, 2000 to July 5th, 2023 and in English, potentially excluding relevant literature. However, to the best of our knowledge, very little literature was published on this topic prior to 2000.

We offer recommendations as this literature continues to grow. Future studies can extend current research by examining maternal/fetal sex steroid hormones as mediators on the pathway between synthetic chemical exposures and child outcomes. Zhang et al. [41] provide an example of that approach by examining sex steroid hormones as mediators in the association between maternal triclosan exposure and offspring outcomes including ponderal index and head circumference [41]. Further, future research can examine critical periods of exposure to determine if disruption at specific points during pregnancy (e.g., early vs. late) has different implications for maternal and fetal health. Repeat measures of exposure across pregnancy, but also within trimester, is particularly important for the non-persistent chemicals, which tend to show low stability over time [61]. For persistent chemicals, future studies might consider associations by parity given the potential for “shedding” of persistent organic pollutants (POPs) through birth and breastfeeding [62, 63]. To date, this literature has relied primarily upon single pollutant models that evaluate each chemical separately. Consistent with general advances in environmental epidemiology, future studies should additionally incorporate analytic approaches to evaluate joint exposure to multiple synthetic chemicals within and across classes (“mixtures”) in relation to maternal hormone profiles. The analysis of additional sex steroid hormones (e.g., dehydroepiandrosterone and dihydrotestosterone) will enrich our understanding of this topic and the mechanisms by which EDCs disrupt steroidogenic pathways. More primary research is specifically required for dioxins, pesticides, and flame retardants, as these classes have been given limited attention in the literature. Conversely, phenols, parabens, phthalates, and PFAS have a richer body of primary evidence, warranting consideration for systematic reviews.

## Conclusion

Overall, the epidemiological research shows that exposures to common synthetic chemicals are associated with sex steroid hormones during pregnancy. However, many of the findings are mixed, with differences in the direction of the estimate, differences by fetal sex, and differences by timing of hormone measurement. As these synthetic chemical classes become more prominent in the environment and in consumer goods, it is important to continue monitoring their impact on the health of pregnant people and their offspring.

### Electronic Supplementary Material

Below is the link to the electronic supplementary material.

Supplementary Material 1

## Data Availability

No datasets were generated or analysed during the current study.

## Abbreviations

BH-FDR: Benjamini-Hochberg false discovery rate
BMI: Body mass index
BPA: Bisphenol A
BPS: Bisphenol S
BP-3: Benzophenone-3
BuPB: Butylparaben
DDT: Dichlorodiphenyltrichloroethane
DEHP: Di(2-ethylhexyl) phthalate
DEP: Diethylphosphate
DETP: Diethylthiophosphate
DMP: Dimethylphosphate
EDCs: Endocrine-disrupting chemicals
EPB: Ethylparaben
E1: Estrone
E2: Estradiol
E3: Estriol
fT: Free testosterone
HCB: Hexachlorobenzene
HCH: Hexachlorocyclohexane
HMWP: High-molecular-weight phthalates
IQR: Interquartile range
MBP: Mono-butyl phthalate
MBzP: Monobenzyl phthalate
MCNP: Mono(carboxynonyl) phthalate
MCOP: Mono-carboxyisooctyl phthalate
MCPP: Mono(3-carboxypropyl) phthalate
MECPP: Mono-2-ethyl-5-carboxypentyl phthalate
MEHHP: Mono-2-ethyl-5-hydroxyhexyl phthalate
MEHHTP: Mono-2-ethyl-5-hydrohexyl terephthalate
MEHP: Mono-(2-ethylhexyl) phthalate
MEOHP: Mono(2-ethyl-5-oxohexyl) phthalate
MEP: Mono-ethyl phthalate
MHBP: Mono-hydroxybutyl phthalate
MiBP: Mono-isobutyl phthalate
mono-o-PCB: Mono ortho polychlorinated biphenyl
MPB: Methylparaben
non-o-PCB: Non ortho polychlorinated biphenyl
OCP: Organochlorine pesticides
OH-MiNP: Mono(hydroxyl-methyl-octyl) phthalate
PBDE: Polybrominated diphenyl ether
PCBs: Polychlorinated biphenyls
PCDDs: Polychlorinated dibenzo-p-dioxins
PCDD/F: Polychlorinated dibenzo-p-dioxins and dibenzofuran
PCDFs: Polychlorinated dibenzofuran
PFAS: Poly- and perfluoroalkyl substances
PFBA: Perfluorobutanoic acid
PFDA: Perfluorodecanoic acid
PFHxS: Perfluorohexane sulfonic acid
PFNA: Perfluorononanoic acid
PFOA: Perfluorooctanoic acid
PFOS: Perfluorooctanesulfonic acid
PFUdA: Perfluoroundecanoic acid
POPs: Persistent organic pollutants
PPB: Propylparaben
p,p’-DDE: Dichlorodiphenyldichloroethylene
T: Testosterone
TCS: Triclosan
TT: Total testosterone
2,4-DCP: 2,4-dichlorophenol
2,5-DCP: 2,5-dichlorophenol
∑DiNP: Molar sum of di-iso-nonyl phthalate metabolites
∑DnOP: Molar sum of di-n-octyl phthalate metabolites
