# Supplementary material for: Exposure to Synthetic Endocrine-Disrupting Chemicals in Relation to Maternal and Fetal Sex Steroid Hormones: A Scoping Review
Source: Curr Environ Health Rep. 2024 Jul 22;11(3):356–79. doi: 10.1007/s40572-024-00455-6 (PMC11324767; doi:10.1007/s40572-024-00455-6)
Supplement: Supplementary file 1 — Supplementary Material 1 [file 40572_2024_455_MOESM1_ESM.docx]

**Supplemental Table 1:** Preferred Reporting Items for Systematic reviews and Meta-Analyses extension for Scoping Reviews (PRISMA-ScR) Checklist

| **SECTION** | **ITEM** | **PRISMA-ScR CHECKLIST ITEM** | **REPORTED ON PAGE #** |
| --- | --- | --- | --- |
| **TITLE** | | | |
| Title | 1 | Identify the report as a scoping review. | 1 |
| **ABSTRACT** | | | |
| Structured summary | 2 | Provide a structured summary that includes (as applicable): background, objectives, eligibility criteria, sources of evidence, charting methods, results, and conclusions that relate to the review questions and objectives. | 2 |
| **INTRODUCTION** | | | |
| Rationale | 3 | Describe the rationale for the review in the context of what is already known. Explain why the review questions/objectives lend themselves to a scoping review approach. | 3 |
| Objectives | 4 | Provide an explicit statement of the questions and objectives being addressed with reference to their key elements (e.g., population or participants, concepts, and context) or other relevant key elements used to conceptualize the review questions and/or objectives. | 3 |
| **METHODS** | | | |
| Protocol and registration | 5 | Indicate whether a review protocol exists; state if and where it can be accessed (e.g., a Web address); and if available, provide registration information, including the registration number. | 4 |
| Eligibility criteria | 6 | Specify characteristics of the sources of evidence used as eligibility criteria (e.g., years considered, language, and publication status), and provide a rationale. | 4-5 |
| Information sources* | 7 | Describe all information sources in the search (e.g., databases with dates of coverage and contact with authors to identify additional sources), as well as the date the most recent search was executed. | 4-5 |
| Search | 8 | Present the full electronic search strategy for at least 1 database, including any limits used, such that it could be repeated. | Supplemental |
| Selection of sources of evidence† | 9 | State the process for selecting sources of evidence (i.e., screening and eligibility) included in the scoping review. | 4-5 |
| Data charting process‡ | 10 | Describe the methods of charting data from the included sources of evidence (e.g., calibrated forms or forms that have been tested by the team before their use, and whether data charting was done independently or in duplicate) and any processes for obtaining and confirming data from investigators. | 5 |
| Data items | 11 | List and define all variables for which data were sought and any assumptions and simplifications made. | 5 |
| Critical appraisal of individual sources of evidence§ | 12 | If done, provide a rationale for conducting a critical appraisal of included sources of evidence; describe the methods used and how this information was used in any data synthesis (if appropriate). | 5 |
| Synthesis of results | 13 | Describe the methods of handling and summarizing the data that were charted. | 5 |
| **RESULTS** | | | |
| Selection of sources of evidence | 14 | Give numbers of sources of evidence screened, assessed for eligibility, and included in the review, with reasons for exclusions at each stage, ideally using a flow diagram. | Figure 1 |
| Characteristics of sources of evidence | 15 | For each source of evidence, present characteristics for which data were charted and provide the citations. | 5-6 |
| Critical appraisal within sources of evidence | 16 | If done, present data on critical appraisal of included sources of evidence (see item 12). | N/A |
| Results of individual sources of evidence | 17 | For each included source of evidence, present the relevant data that were charted that relate to the review questions and objectives. | Tables 1-4 |
| Synthesis of results | 18 | Summarize and/or present the charting results as they relate to the review questions and objectives. | 6-12 |
| **DISCUSSION** | | | |
| Summary of evidence | 19 | Summarize the main results (including an overview of concepts, themes, and types of evidence available), link to the review questions and objectives, and consider the relevance to key groups. | 12-14 |
| Limitations | 20 | Discuss the limitations of the scoping review process. | 14 |
| Conclusions | 21 | Provide a general interpretation of the results with respect to the review questions and objectives, as well as potential implications and/or next steps. | 14 |
| **FUNDING** | | | |
| Funding | 22 | Describe sources of funding for the included sources of evidence, as well as sources of funding for the scoping review. Describe the role of the funders of the scoping review. | 15 |

JBI = Joanna Briggs Institute; PRISMA-ScR = Preferred Reporting Items for Systematic reviews and Meta-Analyses extension for Scoping Reviews.

* Where *sources of evidence* (see second footnote) are compiled from, such as bibliographic databases, social media platforms, and Web sites.

† A more inclusive/heterogeneous term used to account for the different types of evidence or data sources (e.g., quantitative and/or qualitative research, expert opinion, and policy documents) that may be eligible in a scoping review as opposed to only studies. This is not to be confused with *information sources* (see first footnote).

‡ The frameworks by Arksey and O’Malley (6) and Levac and colleagues (7) and the JBI guidance (4, 5) refer to the process of data extraction in a scoping review as data charting*.*

§ The process of systematically examining research evidence to assess its validity, results, and relevance before using it to inform a decision. This term is used for items 12 and 19 instead of "risk of bias" (which is more applicable to systematic reviews of interventions) to include and acknowledge the various sources of evidence that may be used in a scoping review (e.g., quantitative and/or qualitative research, expert opinion, and policy document).

*From:* Tricco AC, Lillie E, Zarin W, O'Brien KK, Colquhoun H, Levac D, et al. PRISMA Extension for Scoping Reviews (PRISMAScR): Checklist and Explanation. Ann Intern Med. 2018;169:467–473. [doi: 10.7326/M18-0850](http://annals.org/aim/fullarticle/2700389/prisma-extension-scoping-reviews-prisma-scr-checklist-explanation).

**Supplemental Table 2:** Search strategies for systematic review of common synthetic endocrine-disrupting chemicals and maternal and fetal sex steroid hormones

| **Database Searching** | | | |
| --- | --- | --- | --- |
| **Databases** | **Search Strategies** | **Filters Applied** | **Date** |
| **PubMed** | ((("Mothers"[Mesh] OR mother[tiab] OR mothers[tiab] OR fetal[tiab] OR "fetus"[Mesh] OR "Pregnancy"[Mesh] OR pregnant[tiab] OR "Pregnant Women"[Mesh])) **AND** (("Gonadal Steroid Hormones"[Mesh] OR "Sex steroid hormones"[tiab] OR (sex steroid) OR "Testosterone"[Mesh] OR Testosterone[tiab] OR "free testosterone" OR "Androgens"[Mesh] OR androgens[tiab] OR "Estrogens"[Mesh] OR estrogen[tiab] OR estrogens[tiab] OR "Estrone"[Mesh] OR "Estradiol"[Mesh] OR "Estriol"[Mesh] OR (sex hormone binding globulin) OR SHBG)))  **AND** (("Maternal Exposure"[Mesh] OR (maternal exposure) OR (Prenatal Exposure) OR prenatal OR pregnancy OR "Endocrine Disruptors"[Mesh] "Endocrine Disruptor"[tiab] OR EDC or (endocrine disrupting chemicals) OR (perfluoroalkyl substances) OR (polyfluoroalkyl substances) OR PFAS OR PFOA OR PFOS OR "parabens"[mesh] OR "parabens"[tiab] OR "paraben"[tiab] OR phthalate OR phthalate[tiab] OR "phthalic acids"[mesh] OR "phthalic acids"[tiab] OR "phenol"[tiab] OR "phenol"[mesh] OR "Bisphenol A" OR BPA OR "triclosan"[mesh] OR "triclosan"[tiab] OR "Dioxins"[Mesh] OR dioxin OR "Dioxins and Dioxin-like Compounds"[Mesh] OR "flame retardants" OR "Polybrominated diphenyl ethers" OR PBDE OR "polybrominated biphenyl" OR PBBs OR HBCDDs OR TBBPA OR "Polychlorinated Biphenyls"[Mesh] OR "Polychlorinated Biphenyls"[TIAB] OR PCBs OR "Pesticides"[Mesh] OR pesticide OR oxybenzone OR benzophenone-3)) | =2,172 results  Limited to English =2,103  Limited to humans  =865  Limited to 2000 to present =**763** results exported | 7/5/2023 |
| **PubMed** (Rerun for updated references) | ((("Mothers"[Mesh] OR mother[tiab] OR mothers[tiab] OR fetal[tiab] OR "fetus"[Mesh] OR "Pregnancy"[Mesh] OR pregnant[tiab] OR "Pregnant Women"[Mesh])) **AND** (("Gonadal Steroid Hormones"[Mesh] OR "Sex steroid hormones"[tiab] OR (sex steroid) OR "Testosterone"[Mesh] OR Testosterone[tiab] OR "free testosterone" OR "Androgens"[Mesh] OR androgens[tiab] OR "Estrogens"[Mesh] OR estrogen[tiab] OR estrogens[tiab] OR "Estrone"[Mesh] OR "Estradiol"[Mesh] OR "Estriol"[Mesh] OR (sex hormone binding globulin) OR SHBG)))  **AND** (("Maternal Exposure"[Mesh] OR (maternal exposure) OR (Prenatal Exposure) OR prenatal OR pregnancy OR "Endocrine Disruptors"[Mesh] "Endocrine Disruptor"[tiab] OR EDC or (endocrine disrupting chemicals) OR (perfluoroalkyl substances) OR (polyfluoroalkyl substances) OR PFAS OR PFOA OR PFOS OR "parabens"[mesh] OR "parabens"[tiab] OR "paraben"[tiab] OR phthalate OR phthalate[tiab] OR "phthalic acids"[mesh] OR "phthalic acids"[tiab] OR "phenol"[tiab] OR "phenol"[mesh] OR "Bisphenol A" OR BPA OR "triclosan"[mesh] OR "triclosan"[tiab] OR "Dioxins"[Mesh] OR dioxin OR "Dioxins and Dioxin-like Compounds"[Mesh] OR "flame retardants" OR "Polybrominated diphenyl ethers" OR PBDE OR "polybrominated biphenyl" OR PBBs OR HBCDDs OR TBBPA OR "Polychlorinated Biphenyls"[Mesh] OR "Polychlorinated Biphenyls"[TIAB] OR PCBs OR "Pesticides"[Mesh] OR pesticide OR oxybenzone OR benzophenone-3)) | =2,207 results  Limited to English =2,138  Limited to humans  =891  Limited to 2023/07/05 to present =**24** results exported | 1/15/2024 |
| **Scopus** | TITLE-ABS-KEY(mothers OR mother OR fetal OR fetus OR "pregnant women" OR "pregnant woman") **AND** TITLE-ABS-KEY("gonadal steroid hormones" OR "sex steroid hormones" OR "sex steroid" OR testosterone OR "free testosterone" OR androgens OR androgen OR estrogens OR estrogen OR estrone OR estradiol OR estriol OR "sex hormone binding globulin" OR SHBG) **AND** TITLE-ABS-KEY("endocrine disruptor" OR EDC OR "endocrine disrupting chemicals" OR "perfluoroalkyl substances" OR "polyfluoroalkyl substances" OR PFAS OR PFOA OR PFOS OR parabens OR paraben OR phthalate OR "phthalic acids" OR phenol OR "Bisphenol A" OR BPA OR triclosan OR dioxins OR dioxin OR "Dioxins and Dioxin-like Compounds" OR "flame retardants" OR "Polybrominated diphenyl ethers" OR PBDE OR "polybrominated biphenyl" OR PBBs OR HBCDDs OR TBBPA OR "Polychlorinated Biphenyls" OR PCBS OR pesticides OR pesticide OR oxybenzone OR benzophenone-3) | =1,859 results  Limited to English =1,779  Limited to 2000 to 2023  =**1,601** results exported | 7/5/2023 |
| **Embase** | (mother:ti,ab,kw OR fetus:ti,ab,kw OR fetal:ti,ab,kw OR 'pregnant woman':ti,ab,kw OR pregnancy:ti,ab,kw)  **AND** ('sex hormone':ti,ab,kw OR 'reproductive hormone':ti,ab,kw OR testosterone:ti,ab,kw OR androgen:ti,ab,kw OR estrogen:ti,ab,kw OR estrone:ti,ab,kw OR estradiol:ti,ab,kw OR estriol:ti,ab,kw OR 'sex hormone binding globulin':ti,ab,kw OR 'sex hormone'/exp OR 'androgen'/exp OR 'estrogen'/exp OR 'testosterone'/exp) **AND**  ('endocrine disruptor':ti,ab,kw OR 'perfluoroalkyl substances' OR 'polyfluoroalkyl substances' OR paraben OR phthalate OR 'phthalic acid' OR phenol OR 'bisphenol a' OR triclosan OR dioxin OR 'dioxins and dioxin-like compounds' OR 'flame retardant' OR 'polybrominated diphenyl ether' OR 'polybrominated biphenyl' OR pesticide OR oxybenzone OR 'endocrine disruptor'/exp OR 'phthalic acid'/exp OR 'phenol'/exp OR 'triclosan'/exp OR 'dioxin'/exp OR 'flame retardant'/exp OR 'polybrominated diphenyl ether'/exp OR 'polybrominated biphenyl'/exp OR 'pesticide'/exp OR 'oxybenzone'/exp) | = 2,241 results  Limited to English =2,150  Limited to 2000 to Present =1,918  Limited to humans =**1,141** results exported | 7/5/2023 |
